# Response rates in patients with schizophrenia and positive symptoms receiving cognitive behavioural therapy: a systematic review and single-group meta-analysis

**DOI:** 10.1186/s12888-018-1964-8

**Published:** 2018-12-04

**Authors:** Irene Bighelli, Maximilian Huhn, Johannes Schneider-Thoma, Marc Krause, Cornelia Reitmeir, Sofia Wallis, Felicitas Schwermann, Gabi Pitschel-Walz, Corrado Barbui, Toshi A. Furukawa, Stefan Leucht

**Affiliations:** 1 Department of Psychiatry and Psychotherapy, Klinikum rechts der Isar, Technische Universität München, Munich, Germany; 2 WHO Collaborating Centre for Research and Training in Mental Health and Service Evaluation, Department of Neuroscience, Biomedicine and Movement Sciences, Section of Psychiatry, University of Verona, Verona, Italy; 3 Department of Health Promotion and Human Behavior, Kyoto University Graduate School of Medicine/School of Public Health, Kyoto, Japan

**Keywords:** Schizophrenia, Cognitive behavioural therapy, Meta-analysis, Response rate

## Abstract

**Background:**

Cognitive behavioural therapy has been used for schizophrenia, but to which extent it is effective is still controversial. Results of existing meta-analyses are of difficult interpretation, because they mainly present effect sizes in the form of standardized mean differences between intervention and control groups based on rating scales, which are of unclear clinical meaning. No meta-analysis has considered the number of patients responding to treatment yet. Based on this ground, we present the first meta-analysis examining the response rates of patients with schizophrenia and positive symptoms to cognitive behavioural therapy.

**Methods:**

We searched multiple databases for randomized controlled trials on psychological interventions of schizophrenia including patients with positive symptoms, and included for this analysis the studies on cognitive behavioural therapy (last search: January 2018). We applied a validated imputation method to calculate the number of responders from rating scales for the outcomes overall symptoms and positive symptoms, based on two criteria, at least 20% and at least 50% reduction from baseline on PANSS or BPRS total scores. Data were pooled in a single-group summary meta-analysis using R software. Additionally, several potential moderators of response to cognitive behavioural therapy were examined by subgroup and meta-regression analyses. The protocol has been registered in PROSPERO (CRD42017067795).

**Results:**

We included 33 studies with a total of 1142 participants receiving cognitive behavioural therapy. On average, 44.5 and 13.2% of the patients reached a 20% (minimally improved) and 50% (much improved) reduction of overall symptoms. Similarly, 52.9 and 24.8% of the patients reached a 20%/50% reduction of positive symptoms. Subgroup and meta-regression analyses revealed a better treatment response in overall symptoms for patients that were not treatment resistant and in studies with researchers’ allegiance. Of borderline significance was the better response in studies employing expert therapists and in patients that were more severely ill at baseline. Blinding of outcome assessor, number of sessions, treatment duration, age and gender were not significant moderators of response.

**Conclusions:**

Our findings suggest that adding cognitive behavioural therapy to pharmacotherapy brings about a minimal improvement in overall symptoms among 44.5% of its recipients. Several study and patients characteristics can moderate response rates.

**Electronic supplementary material:**

The online version of this article (10.1186/s12888-018-1964-8) contains supplementary material, which is available to authorized users.

## Background

Schizophrenia is a severe disorder, and a leading cause of disability with a dramatic burden on society [1]. Psychological interventions for schizophrenia have been developed to address several aspects of the disorder, and in agreement with guidelines from the National Institute for Health and Care Excellence in the UK and the Schizophrenia Patient Outcomes Research Team in the USA, are widely regarded as necessary interventions [2, 3]. The importance of research advancements in the field of psychological treatments has been also recently pointed out by the constitution of the Lancet Psychiatry Commission on psychological treatments research in tomorrow’s science [4].

Among psychotherapies for schizophrenia, cognitive behavioural therapy (CBT) is the most studied, and it is currently recommended by guidelines [2]. In a recent systematic review and network meta-analysis by our group, which considered all psychological interventions for schizophrenia, 41 out of 53 included randomized controlled trials (RCTs) examined CBT [5]. Results revealed that CBT is efficacious for treating patients with schizophrenia who present positive symptoms, which had a significant benefit from the treatment when compared to patients receiving usual care, supportive therapy and inactive control conditions such as befriending. In particular, effect sizes measured as standardized mean differences of CBT in comparison with usual care were − 0.38 (95% CI -0.56 to − 0.20) for overall symptoms, − 0.30 (95% CI -0.30 to − 0.14) for positive symptoms and − 0.16 (95% CI -0.29 to − 0.03) for negative symptoms [5].

However, efficacy measured with rating scales is difficult to interpret. The clinical meaning of results is especially unclear when different measures are used in different studies, and a standardized mean difference is employed as effect size*.* A pragmatic outcome like response to treatment would make the results easier to interpret. Moreover, meta-analyses provide the relative treatment effects in comparison to an alternative intervention, while, from a clinical point of view, it is important to know the absolute treatment effect that can be expected from a certain therapy.

Nonetheless, the number of patients who improve with a treatment is rarely reported in the studies, and very heterogeneous criteria are used to define it. Probably for that reason, not one of the existing pairwise meta-analyses on CBT for schizophrenia presented data on response rates. The only exception is represented by a Cochrane review by Jones et al., in which the authors pooled response rates from seven trials under the label of “reliable change”, pointing out that these trials applied different definitions of response [6]. They presented a pooled relative effect size that did not inform on the absolute treatment effect of cognitive behavioural treatment.

As a result, the extent to which patients with schizophrenia and positive symptoms may benefit from CBT remains unclear.

A possible strategy to deal with this issue was applied by Zhu and colleagues, who calculated response rates from continuous outcomes in the field of antipsychotic medication for patients with first episode schizophrenia [7]. Thus, we decided to apply the same methodology to calculate response rates from studies on CBT that were included in the previous review [5], in order to provide an easy-to-interpret measure of treatment effect.

Goals for present meta-analysis are: i) calculating how well patients with schizophrenia and positive symptoms respond to cognitive behavioural therapy; ii) examining the determinants of response to cognitive behavioural therapy in this population.

## Methods

### Study design and participants

The protocol of the original review was registered in PROSPERO (number CRD42017067795) and published [8]. We included studies in adult individuals with a diagnosis of schizophrenia or related disorders (such as schizophreniform or schizoaffective disorders), presenting current positive symptoms, as defined by inclusion criteria of the trial, with no restrictions on setting, gender or ethnicity. We excluded studies on patients with predominant negative symptoms or concomitant medical or psychiatric illness, and patients at different stages of illness (first episode, at risk of psychosis). Studies were included if at least 80% of the patients had schizophrenia or related disorders (such as schizophreniform or schizoaffective disorders). Following the rules of the Cochrane Schizophrenia group we included trials regardless of the diagnostic criteria used [9], in order to increase representativeness and generalizability.

### Intervention, comparator and outcome

For the current analysis, unlike our previous review, we considered only studies on cognitive behavioural therapy, compared with any non-pharmacological intervention or control condition. Among the included studies cognitive behavioural therapy was administered usually in addition to standard care, which typically included pharmacological treatment. Studies were included in the analysis if they provided data for overall symptoms and/or positive symptoms measured with validated rating scales.

### Search strategy and inclusion criteria

We searched Embase, MEDLINE, PsycINFO, PubMed, WHO International Clinical Trials Registry Platform (ICTRP), ClinicalTrials.gov and Cochrane Collaboration Controlled Trials Register for reports published up to January 2018 for randomized controlled trials that compared CBT with other psychological treatments or with a non-pharmacological control condition in patients with schizophrenia currently presenting positive symptoms. We applied no restrictions for language or publication period. Previous reviews on CBT were also inspected to determine if some studies met our inclusion criteria as well.

### Screening and data extraction

Two reviewers among IB, CR, SW and FS independently inspected all abstracts identified in the searches based on the inclusion criteria. Disagreements were resolved by discussion, and in case of doubts the full paper was retrieved for further inspection. Full articles were obtained for all eligible papers, and were again independently assessed by two reviewers. Disagreements were resolved by discussion, and in case of need, by contacting study authors for further information.

Two of IB, CR, SW and FS independently extracted data from the selected studies, considering main reports, secondary publications and supplementary materials, entered the relevant information into a Microsoft-Access database created especially for this study and assessed risk of bias using the Cochrane risk of bias tool [10, 11]. We contacted authors of included studies published in the last 30 years for missing or additional information about their studies.

### Definitions of response

Response is defined typically in schizophrenia trials as a minimum percentage reduction of the PANSS/BPRS total score from baseline to endpoint. Different cut-offs have been used in the literature to define response (for example at least 20, 25, 30, 40% or 50% improvement [12]). According to equipercentile linking studies comparing PANSS/BPRS scores with simultaneous CGI ratings [13], an improvement of at least 20% corresponds approximately to ‘minimally improved’ as measured with the Clinical Global Impressions of the raters, while 50% reduction from baseline means much improved according to the CGI [14–16].

In studies on psychological interventions, the number of patients reaching “response” is not often reported: only 12 out of 62 trials presented this information in our previous review [5]. In trials included in the present analysis this information was reported in 10 out of 33 studies. Moreover, when number of responders is provided, they are often defined with very heterogeneous criteria, that would not be comparable. In order to obtain a reliable measure of the response rate that could be comparable across studies, we calculated the rate of responders from the scores on continuous scales, using the imputation method proposed originally by Furukawa et al. [17] and replicated [7, 18]. We used this method to estimate number of patients who reached at least 20 and 50% reduction from baseline of rating scales measuring overall symptoms (mainly PANSS and BPRS), based on means and standard deviations at endpoint or change scores from baseline. Our primary outcome was the reduction of at least 20% from baseline in overall symptoms scale, that corresponds to a minimal improvement [14]. Since the efficacy of CBT had already been established in our previous network meta-analysis, we wanted now to determine how many patients benefited from the treatment, and even a small decrease in symptoms was regarded as relevant. Additionally, given the focus on patients with positive symptoms, we also calculated response rates from positive symptoms scales, again for 20 and 50% cut-offs. In the case where a scale had a possible minimum baseline score different from 0 (for example, PANSS rated as 1–7 for each item), the application of this method would result in an underestimation of response rates [12, 19]. Therefore, we subtracted the minimum score of the scales (for example 30 in the case of PANSS total) before imputing the number of responders.

### Data analysis

Unlike from most meta-analyses focusing on comparisons between interventions, the aim of the current meta-analysis was to examine the response rate in a population of patients with schizophrenia receiving CBT. Accordingly, in this case the index is not a between-group difference, but rather, a single-group summary, that uses in essence the same meta-analytic calculations [20]. To obtain an average response rate, we performed a single-group summary meta-analysis in R using the metaprop function in the meta package [21, 22]. Analyses were conducted separately for both outcomes (reduction in overall and positive symptoms), for both cutoffs (at least 20% and at least 50% reduction from baseline), and using the intention-to-treat datasets.

Heterogeneity was assessed using the I-square statistic (values > 50% were considered considerable heterogeneity) [23].

In order to explore which study characteristics might explain heterogeneity, we performed subgroup (dichotomous variables) and meta-regression analyses (continuous variables) for the primary outcome response rate at 20% reduction in overall symptoms. When the analysis revealed a possible role for a specific moderator, we investigated further on the number of responders calculated with a 50% reduction in overall symptoms threshold. The following moderators were chosen a priori: blinding of outcome assessor, treatment resistant patients, researchers’ allegiance (whether study authors also developed the investigational intervention of the study), expertise of the therapist, number of sessions, treatment duration, baseline severity, mean age, gender ratio and percentage of participants taking antipsychotic medication. We assessed small-study effects by visual examination of funnel plots.

## Results

### Description of included studies

**Fig. 1:**
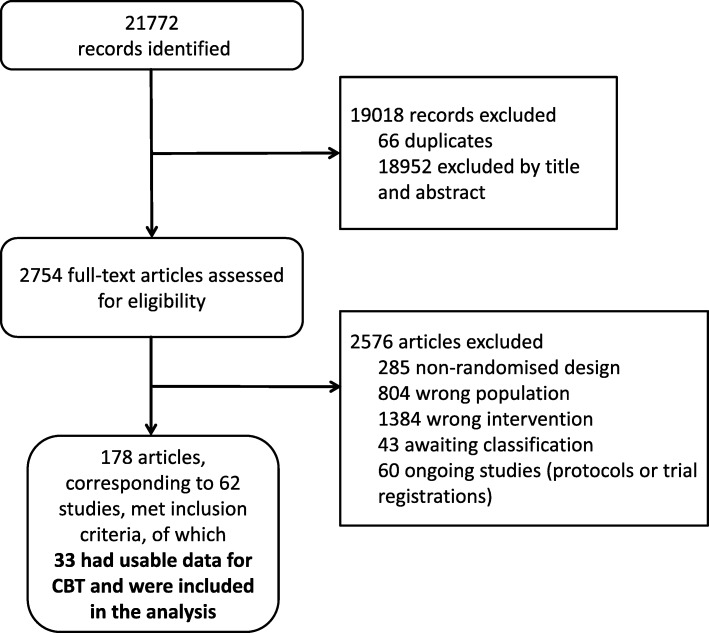
Study selection process

We identified 21,772 unique references through the literature search (last update January 2018), of which 2754 were considered eligible after screening of title and abstract. After inspection of full-text, we included 62 randomised controlled trials, of which 33 had usable data and were included in the analyses, with a total of 1142 participants in the CBT arms. The PRISMA flow-chart is presented in Fig. 1. Characteristics and detailed references of included studies are presented in Additional file 1.

For 26 studies it was possible to calculate number of responders from an overall symptoms scale, while for 29 studies it was possible to calculate number of responders from a scale measuring positive symptoms.

Twelve studies enrolled treatment-resistant patients. Treatments were generally delivered by expert therapists (20 studies), while three studies employed therapists in training. The median number of sessions was 12.95, and the median treatment duration 23 weeks (range 4–39). In 21 studies CBT was delivered by a psychologist, in 12 studies by a nurse, and in 7 studies by a psychiatrist. Twelve studies involved different professional figures to deliver CBT, while 6 studies did not provide any information on the professional background of the therapist. In 23 studies the therapists received a specific training for the CBT protocol used in the trial.

The mean age of participants was 37.34 years, and the mean percentage of male participants in each study was 61.1%. The mean baseline severity (PANSS equivalents) was 70.55. Figures illustrating risk of bias assessment are presented in Additional file 2. Overall, the reports often did not provide details on randomization procedures and allocation concealment. As expected in studies on psychological treatments, patients and personnel were never blind to treatment allocation, but twenty-six studies employed a blind rater to assess the outcome. Attrition bias was high in most of the studies, with intention-to-treat data used rarely for analysis. In 21 studies the authors evaluated the efficacy of a treatment that they had developed or manualized, being rated as high risk for researchers’ allegiance. There were no important other biases which would have been relevant for our research question.

### Response rates

**Fig. 2:**
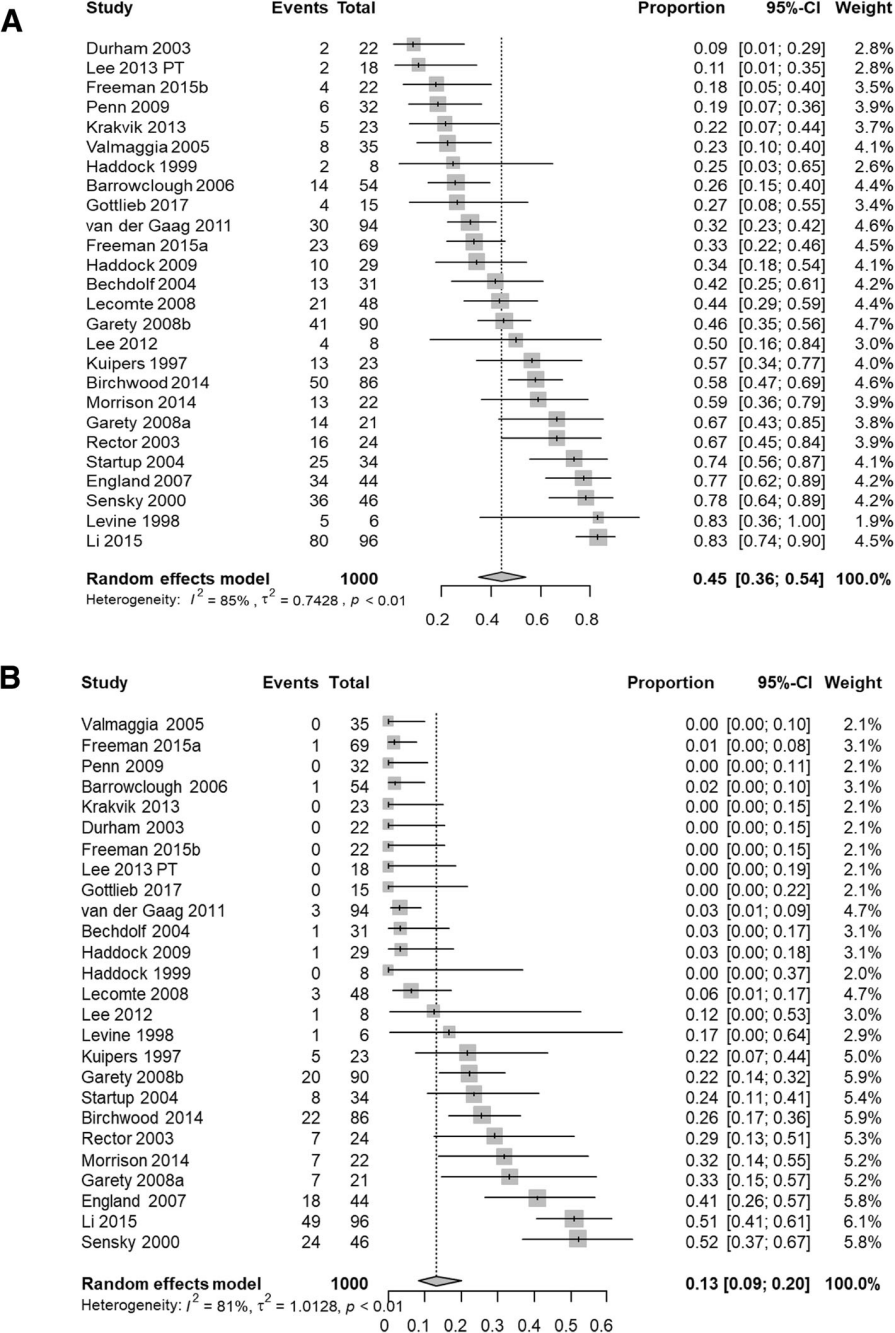
Response rates in overall symptoms. Pooled results for response rates calculated as 20% (**a**) and 50% (**b**) reduction from baseline in overall symptoms

**Fig. 3:**
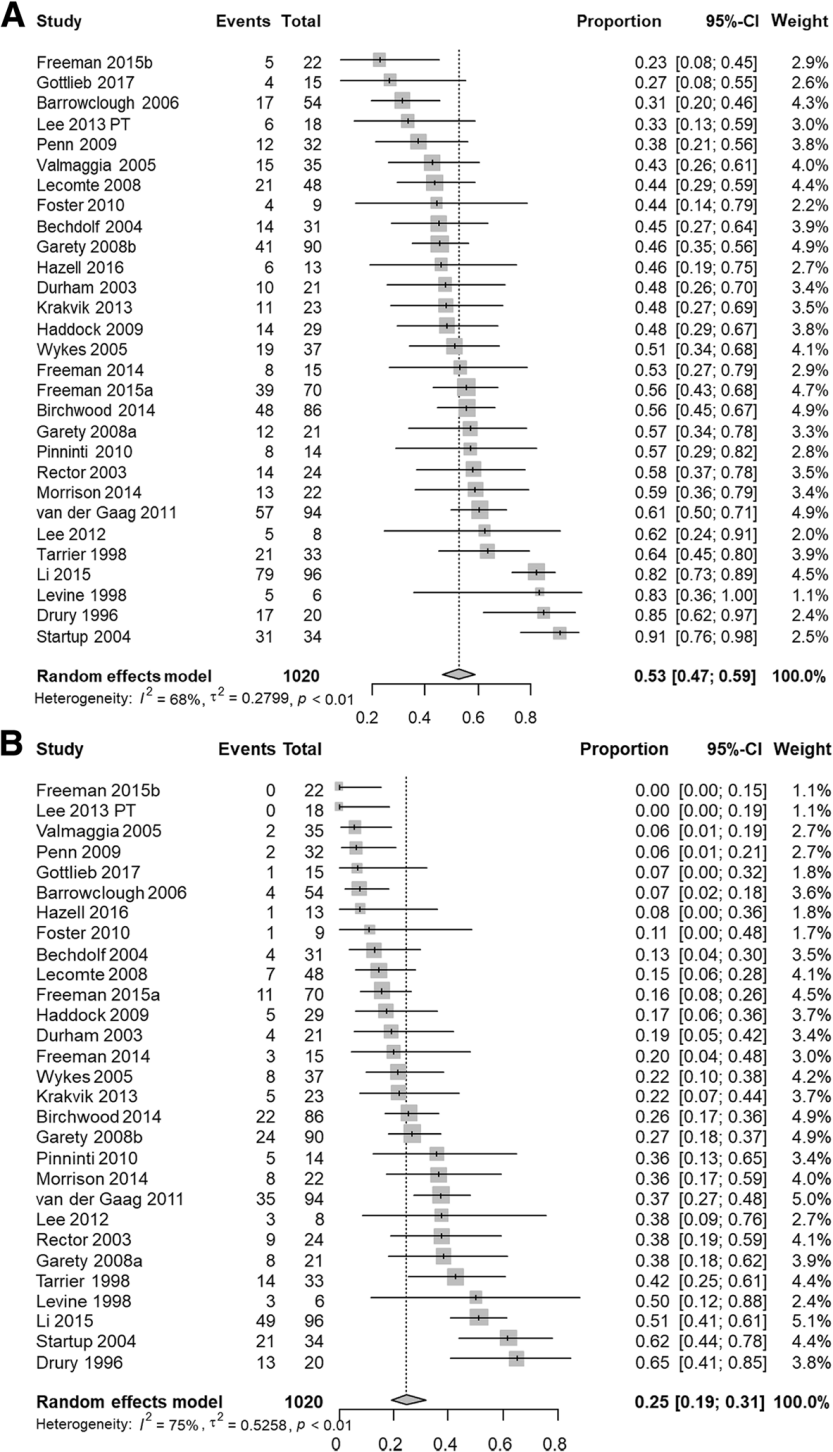
Response rates in positive symptoms. Pooled results for response rates calculated as 20% (**a**) and 50% (**b**) reduction from baseline in positive symptoms

The pooled response rate for the cutoff at least 20% reduction from baseline in overall symptoms was 44.5% (26 RCTs, 1000 participants, 95% CI 35.5 to 53.9%, I^2^ = 85%), and the pooled response rate for the cutoff of at least 50% reduction from baseline was 13.2% (26 RCTs, 1000 participants, 95% CI 8.5 to 20.0%, I^2^ = 81%) (Fig. 2). When considering positive symptoms scales, the pooled response rate for the 20% cutoff was 52.9% (29 RCTs, 1020 participants, 95% CI 46.7 to 59%, I^2^ = 68%), and the pooled response rate for the 50% cutoff was 24.8% (29 RCTs, 1020 participants, 95% CI 19.1 to 31.5%, I^2^ = 75%) (Fig. 3). All the analyses revealed a considerable heterogeneity in the response rates between the different studies, which we explored in subgroup and meta-regression analyses.

### Subgroup and meta-regression analyses (Table 1 and Table 2)

#### Blinded vs open label studies

The test for subgroup differences of response rate between rater-blinded studies and open-label studies was not statistically significant (42.6% vs 50.9%, *p* = 0.6238).

**Table 1:** Subgroup analyses (dichotomous moderators) – 20% overall symptoms reduction

					Test for subgroup differences	
Moderator		N	Responders rate	95% CI	Q	*P*-value
Blinding of outcome assessment	Blind	22	0.4263	0.33; 0.53	0.24	0.62
	Open	3	0.5086	0.23; 0.79		
Treatment resistant	Yes	11	0.3338*	0.22; 0.47	4.75	0.03*
	No	15	0.5327*	0.42; 0.64		
Researchers’ allegiance	Yes	17	0.5113*	0.40; 0.62	4.38	0.04*
	No	9	0.3275*	0.22; 0.46		
Therapist’s expertise	Expert	15	0.4853	0.37; 0.60	3.15	0.07
	Trainee	3	0.3006	0.17; 0.47		

*p < 0.05

**Table 2:** Meta-regression analyses (continuous moderators) - 20% overall symptoms reduction

Moderator	Coefficient	95% CI	Z value	*P*-value
Number of sessions	0.0059	−0.02; 0.03	0.43	0.67
Treatment duration	0.0022	−0.007; 0.01	0.45	0.65
Baseline severity	0.0113	−0.0003; 0.0228	1.92	0.05
Mean age	0.0117	−0.005; 0.03	1.41	0.16
Male percentage	0.3081	−0.16; 0.77	1.29	0.20

#### Treatment-resistant vs other patients

We found a statistically significant lower response rate in studies in patients that did not respond to a previous treatment compared to studies in patients that were not treatment resistant (33.4% vs 53.3%, *p* = 0.0293).

When looking at a 50% symptom reduction cutoff, we found that treatment-resistant patients had a 6.5% response rate, compared to the others who responded in 18.8% of cases (*p* = 0.0553, not shown in the table).

#### Researchers’ allegiance

We found a statistically significant higher response rate in studies in which authors evaluated the therapy that they developed (51.1% vs 32.8%, *p* = 0.0363).

Also when considering the 50% threshold, researchers’ allegiance had a significant impact on the responders rate (20.7% vs 4.9%, *p* = 0.0026, not shown in the table).

#### Expertise of the therapist

The response rate in studies that employed expert therapists was 48.5%, while in studies allowing trainees as therapists it was 30%. However, this difference was only of borderline significance (*p* = 0.0758). We further examined the effect of this moderator on the percentage of patients who obtained a 50% reduction of symptoms, and found a significant effect difference between the two groups (responders rate with expert therapists 18.9%, with trainees 4.5%, *p* = 0.0056, not shown in the table).

#### Number of sessions

Response rate was not found to be associated with number of sessions (*p* = 0.6690).

#### Treatment duration

We did not find a role of study duration in moderating response rates (*p* = 0.6530).

#### Baseline severity – Overall symptoms

We found that baseline severity could have a role in moderating response rates, even if of borderline significance (*p* = 0.0552). When further investigating the effect of baseline severity on the percentage of patients who obtained a 50% reduction of symptoms, there was no effect for this moderator (*p* = 0.174, not shown in the table).

#### Mean age

Response rates were not found to be associated with patients’ mean age (*p* = 0.1581).

#### Percentage male participants

Response rates were not found to be associated with percentage of males (*p* = 0.1952).

#### Percentage of participants taking antipsychotics

Information about number of patients actually receiving medication with antipsychotics was very seldom given in the trials, and never separately for the different arms. Therefore, it was not possible to investigate the role of concurrent antipsychotic medication as a moderator of response.

#### Small study effects

There was no obvious asymmetry in the funnel plot that would have indicated small-study effects. This was confirmed by Egger’s test for forest plot asymmetry (*p* = 0.4167) (see Additional file 3).

## Discussion

To the best of our knowledge, this is the first systematic review that informs on how well patients with schizophrenia and current positive symptoms respond to cognitive behavioural therapy in randomized trials.

Our main findings were that 44.5% of patients who received CBT reached an at least 20% reduction from baseline in overall symptoms, and can be considered at least minimally improved, while 13.2% of patients reached an at least 50% reduction from baseline in overall symptoms, being considered much improved [14]. A decrease in positive symptoms of at least 20%/50% occurred in 52.9%/24.8% of patients, respectively. The observed improvement in positive symptomatology might be explained with the fact that CBT for psychosis actively addresses the thoughts and cognitions related to delusions and hallucinations.

We also found that the patients’ characteristics of being treatment resistant, the severity at baseline, and the clinician’s factors of researchers’ allegiance and expertise could have a role as determinants of response to cognitive behavioural therapy.

The response rates were lower in treatment-resistant patients, who failed to benefit from a previous treatment. This finding may be explained by the fact that treatment-resistant symptoms are more difficult to treat, and therefore a CBT intervention can bring only a lower improvement compared to that of other patients. A trial on clozapine-resistant patients receiving CBT, published after the date of our search, reported responders’ rates that are slightly higher than the ones that we found (46 and 7% for the 20 and 50% PANSS total reduction of symptoms, respectively) [24].

We found a borderline significance for higher response rates in more severely ill patients. This is consistent with previous findings, in which more severely ill patients at baseline had a higher response rate with antipsychotics than less severely ill patients [25, 26].

A reason for the higher response rates in studies conducted by researchers testing the efficacy of their own treatments could be that they might have a vested interest in showing better results for cognitive behavioural therapy. In order to assess the role of this factor, studies should always report information on researchers’ allegiance.

We also found that patients treated by expert therapists had higher response rates, especially when considering the 50% reduction from baseline threshold. The expertise of the therapist might play a more important role when aiming to achieve a greater symptom reduction.

We did not find a role for the other variables that we investigated as possible moderators (blinding type, number of sessions, treatment duration, age and gender).

It must be noted that, on average, patients in the included studies were only moderately ill, with a baseline PANSS total of 70.55, that corresponds to a CGI between 3 and 4 [14], and is importantly lower than the one of patients enrolled in antipsychotics trials [27].

Some limitations should be considered in interpreting our results.

First, it has been shown that the imputation method of response data tends to overestimate very low values and to underestimate extremely high values [18]. In the case of the present analysis, we adopted a conservative approach in our calculations and subtracted the minimum scores only where it was explicitly declared that the 1–7 version of PANNS and BPRS was used. This may have led to a certain degree of imprecision in calculating the response rates. Future studies should always clearly report which version of BPRS / PANSS was employed in order to allow more precise calculations.

Second, patients in the included studies were also receiving standard care, which usually included antipsychotics, so that cognitive behavioural therapy was delivered as add-on to the pharmacological treatment. However, detailed information on antipsychotic medication was usually not provided in the studies. As a result, it is not possible to ascertain the respective role of cognitive behavioural therapy and medication on the outcome, neither to evaluate the adequacy of the pharmacotherapy provided in combination with CBT. Moreover, administration of CBT to patients with schizophrenia without concomitant antipsychotic medication is a debated issue: some studies have been conducted by Morrison et al. in patients receiving CBT without medication [24, 28, 29], but other authors have claimed this to be unethical [30]. We argue that the situation in the studies included in the present review resembles real-life clinical practice settings, where patients, in general, receive antipsychotics in addition to CBT, making our results more generalizable to the clinical context. We claim that future trials should provide detailed information on antipsychotic medication, such as number of patients who actually received antipsychotics and dosages, so that the role of medication can be assessed and differentiated from the role of cognitive behavioural therapy.

A further weakness of these results is the high heterogeneity that we found across different studies. However, we found possible explanations in the role of different moderators as possible sources for heterogeneity in response rates.

This study also presents some strengths. First, the study was planned carefully in agreement with PRISMA guidelines, and followed a sound methodology that was a-priori published in the protocol, including a comprehensive search and the evaluation of quality of studies with the Cochrane Risk of Bias tool. Second, results presented as response rates are easy to interpret for clinicians, and can provide, at first glance, information on the patients’ probability of receiving a benefit from CBT. This information, together with the relative effect sizes coming from comparison of CBT with control conditions, can provide a more complete picture to be considered in the decision making process of treatment strategies for patients with schizophrenia.

## Conclusions

We conclude that adding CBT to pharmacotherapy brings about a minimal improvement in overall symptoms among 44.5% of its recipients, and a considerable improvement among 13.2%. This seems to be particularly relevant for patients that are not treatment resistant, who are more severely ill at baseline and when the treatment is provided by expert therapists. Clinicians can expect a benefit within this order of magnitude when considering offering cognitive behavioural therapy to patients with schizophrenia and positive symptoms.

## Additional files

Additional file 1: Included studies (PDF 461 kb)

Additional file 2: Risk of bias assessment (PDF 766 kb)

Additional file 3: Small study effect and publication bias (PDF 402 kb)

## Abbreviations

BPRS: Brief Psychiatric Rating Scale
CBT: Cognitive Behavioural Therapy
PANSS: Positive and Negative Syndrome Scale
