# Supplementary material for: Response rates in patients with schizophrenia and positive symptoms receiving cognitive behavioural therapy: a systematic review and single-group meta-analysis
Source: BMC Psychiatry. 2018 Dec 4;18:380. doi: 10.1186/s12888-018-1964-8 (PMC6280425; doi:10.1186/s12888-018-1964-8)
Supplement: Supplementary file 1 — Included studies (PDF 461 kb) [file 12888_2018_1964_MOESM1_ESM.pdf]

Characteristics of included studies

	Country	Study treatments (number of patients)	Trial duration (weeks)	Number of sessions	Study design	Characteristics of patients
Barrowclough 2006 (1)	UK	Cognitive Behavioural Therapy (n=57), TAU (n=56)	26	10.4	SB	82 (73%) men, 31 women (27%); mean age 38.83 years; baseline PANSS total score 63.8, positive symptoms 17.4, negative symptoms 14.1; duration of illness 13.67 years
Bechdolf 2004 (2)	Germany	Cognitive Behavioural Therapy (n=40), Psychoeducation (n=48)	8	11.9	SB	Inpatients; 40 (45%) men, 48 women (55%); mean age 31.8 years; baseline PANSS total score 63.75, positive symptoms 14.35, negative symptoms 16.95; duration of illness 4.45 years
Birchwood 2014 (3)	UK	Cognitive Behavioural Therapy (n=98), TAU (n=99)	39	19	SB	113 (57%) men, 84 women (43%); mean age 37.35 years; baseline PANSS total score 71.73, positive symptoms 19.38, negative symptoms 16.02; duration of illness 15.21 years
Drury 1996 (4)	UK	Cognitive Therapy[1] (n=30), Recreation and support (n= 32)	12	NA	OL	Inpatient; 25 (63%) men, 15 women (37%); mean age 30.7 years; baseline PAS positive symptoms score 6; duration of illness 6.15 years
Durham 2003 (5)	Scotland	Cognitive Behavioural Therapy (n=22), Supportive Therapy (n=23), TAU (n=21)	39	20	SB	Inpatients and outpatients; 45 (68%) men, 21 women (32%); mean age 36.3 years; baseline PANSS total score 96.63, PSYRATS total 35.57; duration of illness 13 years
England 2007 (6)	NA (author's affiliation in Canada)	Cognitive nursing intervention[1] (n=44), TAU (n=21)	18	12	SB	Outpatient; mean age 41 years; baseline BPRS-18 total score 51.05
Foster 2010 (7)	UK	Cognitive Behavioural Therapy (n= 12), TAU (n=12)	4	4	OL	Inpatients and outpatients; 14 (58%) men, 10 women (42%); mean age 36.3 years; baseline PSYRATS total score 35.57
Freeman 2014 (8)	UK	Cognitive Behavioural Therapy (n=15), TAU (n=15)	8	6	SB	Outpatients; 20 (67%) men, 10 women (33%); mean age 41.5 years; baseline PSYRATS total score 18.25
Freeman 2015a (9)	UK	Cognitive Behavioural Therapy (n=73), TAU (n=77)	8	5.5	SB	Inpatients and outpatients; 86 (57%) men, 64 women (43%); mean age 36.3 years; baseline PANSS total score 41.5, PSYRATS total 18.35
Freeman 2015b (10)	UK	Cognitive Behavioural Therapy (n=24), TAU (n=26)	12	7.3	SB	Outpatients; 34 (68%) men, 16 women (32%); mean age 40.9 years; baseline PANSS total score 81.65, baseline PSYRATS total 41.6
Garety 2008a (11)	UK	Cognitive Behavioural Therapy (n=27), Family Intervention (n=28),	39	13.9	SB	Inpatients and outpatients; 60 (72%) men, 23 (28%) women; mean age 36.4 years; baseline PANSS total score 67.31, positive symptoms 17.16, negative symptoms 15.58;

		TAU (n=28)				duration of illness 11.57 years
Garety 2008b (11)	UK	Cognitive Behavioural Therapy (n=106), TAU (n=112)	39	14.3	SB	Inpatients and outpatients; 151 (69%) men, 67 (31%) women; mean age 38.1 years; baseline PANSS total score 64.29, positive symptoms 18.51, negative symptoms 12.38; duration of illness 10.4 years
Gottlieb 2017 (12)	USA	Cognitive Behavioural Therapy (n=19), TAU (n=18)	24	10	SB	Outpatients; 23 (62%) men, 14 women (38%); mean age 42.04 years; baseline BPRS-24 total score 54.92, PSYRATS 53.06, BPRS negative symptoms 6.23
Haddock 1999 (13)	UK	Cognitive Behavioural Therapy (n=10), Supportive counselling (n=11)	5	10.2	SB	Inpatients; 19 (90%) men, 2 women (10%); mean age 29.05 years; baseline BPRS total score 53.1
Haddock 2009 (14)	UK	Cognitive Behavioural Therapy (n=38), Social Activity Therapy (n=39)	26	17	SB	Inpatients and outpatients; 66 (86%), 11 (14%) women; mean age 34.8 years; baseline PANSS total score 63.81, positive symptoms 27.6, negative symptoms 13.04
Hazell 2016 (15)	England	Cognitive Behavioural therapy (n=15), Waitlist (n=15)	12	8	SB	Only protocol, authors confirmed that inclusion criteria are met and provided some outcome data, but no descriptive of patients are available in detail
Krakvik 2013 (16)	Norway	Cognitive Behavioural Therapy (n=23), Waitlist (n=22)	26	20	OL	Inpatients and outpatients; 29 (64%) men, 16 (36%) women; mean age 36.35 years; baseline PANSS total score 60.2, positive symptoms 16.05, negative symptoms 11.75
Kuipers 1997 (17)	UK	Cognitive Behavioural Therapy (n=28), TAU (n=32)	39	18.6	OL	Inpatients and outpatients; 38 (63%) men, 22 (37%) women; mean age 25.45 years; duration of illness 13.05 years
Lecomte 2008 (18)	Canada	Cognitive Behavioural Therapy (n=48), Social Skills Training (n=54), WL (n=27)	13	24	SB	Outpatients; 93 (72%) men, 36 (28%) women; mean age 24.49 years; baseline BPRS-E total score 41.67, BPRS positive symptoms 2, negative symptoms 1.8; duration of illness 2.69
Lee 2012 (19)	South Korea	Cognitive behavioural Social Skills Training[1] (n=12), TAU (n=13)	7	12	SB	Inpatients; 8 (40%) men, 12 (60%) women; mean age 52.22 years; baseline BPRS total score 33.28, SAPS positive symptoms 42.24, SANS negative symptoms 50.05; duration of illness 19.19 years
Lee 2013 (20)	South Korea	Cognitive Behavioural Therapy (n=25), Supportive Therapy (n=25)	32	20.1	SB	Inpatients and outpatients; 21 (57%) men, 16 (43%) women; mean age 41.25 years; baseline PANSS total score 61.25, positive symptoms 18.95, negative symptoms 13.85; duration of illness 15.8 years
Levine 1998 (21)	NA (author's affiliation in Israel)	Cognitive Therapy[1] (n=6), Supportive Therapy (n=6)	6	6	NA	12 (100%) men; mean age 34.5 years; baseline PANSS total score 57.95, positive symptoms 15.45, negative symptoms 13.3; duration of illness 10.92 years
Li 2015 (22)	China	Cognitive Behavioural Therapy (n=96),	24	15	SB	Inpatients and outpatients; 72 (38%) men, 120 (63%) women; mean age 31.36 years; baseline PANSS total score

		Supportive Therapy (n=96)				72.6, positive symptoms 23.43, negative symptoms 20.4; duration of illness 8.21
Morrison 2014 (23)	UK	Cognitive Therapy[1] (n=37), TAU (n=37)	39	13.3	SB	39 (53%) men, 35 (47%) women; mean age 31.32 years; baseline PANSS total score 71.76, positive symptoms 20.98, negative symptoms 14.52
Penn 2009 (24)	USA	Cognitive Behavioural Therapy (n=32), Supportive Therapy (n=33)	12	8.3	SB	Outpatients; 33 (51%) men, 32 (49%) women; mean age 40.65 years; baseline PANSS total score 61.75, positive symptoms 17.55, negative symptoms 13.9; duration of illness 15.4 years
Pinninti 2010 (25)	USA	Cognitive Behavioural Therapy (n=18), TAU (n=15)	12	11.93	SB	Outpatients; 11 (44%) men, 14 (56%) women; mean age 40.49 years; baseline PSYRATS positive symptoms score 32.3; duration of illness 21.4 years
Rector 2003 (26)	Canada	Cognitive Behavioural Therapy (n=24), TAU (n=21)	26	20	SB	Outpatients; 20 (48%) men, 22 (52%) women; mean age 51.6 years; baseline PANSS total score 63.4, positive symptoms 14.8, negative symptoms 15.95; duration of illness 15.1 years
Sensky 2000 (27)	UK	Cognitive Behavioural Therapy (n=46), Befriending (n=44)	39	19	SB	53 (59%) men, 37 (41%) women; mean age 39.5 years; baseline CPRS schizophrenia change scale total score 10.7, SANS 33.45; duration of illness 14.5 years
Startup 2004 (28)	UK	Cognitive Behavioural Therapy (n=47), TAU (n=43)	26	12.9	OL	Inpatients; 68 (76%) men, 22 (24%) women; mean age 30.8 years; baseline BPRS-16 total score 45.75, SAPS positive symptoms 10.7, SANS negative symptoms 8.9; duration of illness 6.95 years
Tarrier 1998 (29)	UK	Cognitive Behavioural Therapy (n=33), Supportive Counselling (n=26), TAU (n=28)	10	20	SB	54 (75%) men, 18 (25%) women; mean age 39.54 years; baseline BPRS hallucinations positive symptoms 21.4, SANS negative symptoms 11.55; duration of illness 14.14 years
Valmaggia 2005 (30)	Netherlands, Belgium	Cognitive Behavioural Therapy (n=36), Supportive Counselling (n=26)	23	16	SB	Inpatients; 41 (71%) men, 17 (29%) women; mean age 35.48 years; baseline PANSS total score 65.4, positive symptoms 17.85, negative symptoms 13.91; duration of illness 9.2 years
van der Gaag 2011 (31)	Netherlands	Cognitive Behavioural Therapy (n=110), TAU (n=106)	26	13	SB	153 (71%) men, 63 (29%) women; mean age 36.99 years; baseline PANSS total score 69.3, PSYRATS total 31.35; duration of illness 10.58 years
Wykes 2005 (32)	UK	Cognitive Behavioural Therapy (n=45), TAU (n=40)	10	7	OL	Outpatients; 50 (59%) men, 35 (41%) women; mean age 39.7 years; baseline PSYRATS hallucination score 27.95

[1] based on the description of the intervention, considered CBT in the analyses. TAU=Treatment as usual, WL=Waitlist, OL=open label, SB=single blind, NA=not available, PANSS=Positive and Negative Syndrome Scale, SAPS=Scale for the Assessment of Positive Symptoms, PSYRATS=Psychotic Symptoms Rating Scale, SANS=Scale for the Assessment of Negative Symptoms, PAS=Psychiatric Assessment Scale

References

1. Barrowclough C, Haddock G, Lobban F, Jones S, Siddle R, Roberts C et al. Group cognitive-behavioural therapy for schizophrenia. Randomised controlled trial. Br J Psychiatry 2006; 189:527–32.

2. Bechdolf A, Knost B, Kuntermann C, Schiller S, Klosterkotter J, Hambrecht M et al. A randomized comparison of group cognitive-behavioural therapy and group psychoeducation in patients with schizophrenia.[Erratum appears in Acta Psychiatr Scand. 2004 Dec;110(6):483]. Acta Psychiatr Scand 2004; 110(1):21–8.

3. Birchwood M, Michail M, Meaden A, Tarrier N, Lewis S, Wykes T et al. Cognitive behaviour therapy to prevent harmful compliance with command hallucinations (COMMAND): A randomised controlled trial. The Lancet Psychiatry 2014; 1(1):23–33.

4. Drury V, Birchwood M, Cochrane R, Macmillan F. Cognitive therapy and recovery from acute psychosis: a controlled trial. I. Impact on psychotic symptoms. Br J Psychiatry 1996; 169(5):593–601.

5. Durham RC, Guthrie M, Morton RV, Reid DA, Treliving LR, Fowler D et al. Tayside-Fife clinical trial of cognitive-behavioural therapy for medication-resistant psychotic symptoms. Results to 3-month follow-up. Br J Psychiatry 2003; 182:303–11. 6. England M. Efficacy of cognitive nursing intervention for voice hearing. Perspectives in psychiatric care 2007; 43(2):69–76.

7. Foster C, Startup H, Potts L, Freeman D. A randomised controlled trial of a worry intervention for individuals with persistent persecutory delusions. Journal of behavior therapy and experimental psychiatry 2010; 41(1):45–51.

8. Freeman D, Pugh K, Dunn G, Evans N, Sheaves B, Waite F et al. An early Phase II randomised controlled trial testing the effect on persecutory delusions of using CBT to reduce negative cognitions about the self: the potential benefits of enhancing self confidence. Schizophr Res 2014; 160(1-3):186–92.

9. Freeman D, Dunn G, Startup H, Pugh K, Cordwell J, Mander H et al. Effects of cognitive behaviour therapy for worry on persecutory delusions in patients with psychosis (WIT): a parallel, single-blind, randomised controlled trial with a mediation analysis. Lancet Psychiatry 2015; 2(4):305–13.

10. Freeman D, Waite F, Startup H, Myers E, Lister R, McInerney J et al. Efficacy of cognitive behavioural therapy for sleep improvement in patients with persistent delusions and hallucinations (BEST): a prospective, assessor-blind, randomised controlled pilot trial. Lancet Psychiatry 2015; 2(11):975–83.

11. Garety PA, Fowler DG, Freeman D, Bebbington P, Dunn G, Kuipers E. Cognitive--behavioural therapy and family intervention for relapse prevention and symptom reduction in psychosis: randomised controlled trial. Br J Psychiatry 2008; 192(6):412–23.

12. Gottlieb JD, Gidugu V, Maru M, Tepper MC, Davis MJ, Greenwold J et al. Randomized Controlled Trial of an Internet Cognitive Behavioral Skills-Based Program for Auditory Hallucinations in Persons With Psychosis. Psychiatric rehabilitation journal 2017; (Pagination):No Pagination Specified.

13. Haddock G, Tarrier N, Morrison AP, Hopkins R, Drake R, Lewis S. A pilot study evaluating the effectiveness of individual inpatient cognitive-behavioural therapy in early psychosis. Soc Psychiatry Psychiatr Epidemiol 1999; 34(5):254–8.

14. Haddock G, Barrowclough C, Shaw JJ, Dunn G, Novaco RW, Tarrier N. Cognitive-behavioural therapy v. social activity therapy for people with psychosis and a history of violence: randomised controlled trial. Br J Psychiatry 2009; 194(2):152–7.

15. Hazell CM, Hayward M, Cavanagh K, Jones AM, Strauss C. Guided self-help cognitive behavioral intervention for VoicEs (GiVE): study protocol for a pilot randomized controlled trial. Trials 2016; 17(1):351.

16. Krakvik B, Grawe RW, Hagen R, Stiles TC. Cognitive behaviour therapy for psychotic symptoms: a randomized controlled effectiveness trial. Behav 2013; 41(5):511–24.

17. Kuipers E, Garety P, Fowler D, Dunn G, Bebbington P, Freeman D et al. London-East Anglia randomised controlled trial of cognitive-behavioural therapy for psychosis. I: effects of the treatment phase. Br J Psychiatry 1997; 171:319–27.

18. Lecomte T, Leclerc C, Corbiere M, Wykes T, Wallace CJ, Spidel A. Group cognitive behavior therapy or social skills training for individuals with a recent onset of psychosis? Results of a randomized controlled trial. J Nerv Ment Dis 2008; 196(12):866–75.

19. Lee DH, Ko SM, Choi YS, Kim KJ, Park H. A Randomized Controlled Pilot Study of Cognitive Behavioral Social Skills Training (Korean version) for Middle- or Older-Aged Patients with Schizophrenia: A Pilot Study. Journal of Korean Neuropsychiatric Association 2012; 51(4):192–201.

20. Lee DE, Lee HJ, Yoon OS, Choi IY, Jo JB, Kang KJ. The Effect of Cognitive Behavioral Therapy in Drug-Resistant Patients with Schizophrenia. Journal of Korean Neuropsychiatric Association 2013; 52(1):26–32.

21. Levine J, Barak Y, Granek I. Cognitive Group Therapy for Paranoid Schizophrenics: Applying Cognitive Dissonance. Journal of Cognitive Psychotherapy 1998; 12(1):3–12.

22. Li ZJ, Guo ZH, Wang N, Xu ZY, Qu Y, Wang XQ et al. Cognitive-behavioural therapy for patients with schizophrenia: a multicentre randomized controlled trial in Beijing, China. Psychol Med 2015; 45(9):1893–905.

23. Morrison AP, Turkington D, Pyle M, Spencer H, Brabban A, Dunn G et al. Cognitive therapy for people with schizophrenia spectrum disorders not taking antipsychotic drugs: A single-blind randomised controlled trial. The Lancet 2014; 383(9926):1395–403.

24. Penn DL, Meyer PS, Evans E, Wirth RJ, Cai K, Burchinal M. A randomized controlled trial of group cognitive-behavioral therapy vs. enhanced supportive therapy for auditory hallucinations. Schizophr Res 2009; 109(1-3):52–9.

25. Pinninti NR, Rissmiller DJ, Steer RA. Cognitive-behavioral therapy as an adjunct to second-generation antipsychotics in the treatment of schizophrenia. Psychiatric services (Washington, D.C.) 2010; 61(9):940–3.

26. Rector N, Seeman M, Segal Z. Cognitive therapy of schizophrenia: Toronto trial. 153rd Annual Meeting of the American Psychiatric Association Chicago, Illionois, USA May 13th-18th 2000 2000.

27. Sensky T, Turkington D, Kingdon D, Scott JL, Scott J, Siddle R et al. A randomized controlled trial of cognitive-behavioral therapy for persistent symptoms in schizophrenia resistant to medication. Arch Gen Psychiatry 2000; 57(2):165–72.

28. Startup M, Jackson MC, Evans KE, Bendix S. North Wales randomized controlled trial of cognitive behaviour therapy for acute schizophrenia spectrum disorders: two-year follow-up and economic evaluation. Psychol Med 2005; 35(9):1307–16.

29. Tarrier N, Yusupoff L, Kinney C, McCarthy E, Gledhill A, Haddock G et al. Randomised controlled trial of intensive cognitive behaviour therapy for patients with chronic schizophrenia. Bmj 1998; 317(7154):303–7.

30. Valmaggia LR, van der Gaag M, Tarrier N, Pijnenborg M, Slooff CJ. Cognitive-behavioural therapy for refractory psychotic symptoms of schizophrenia resistant to atypical antipsychotic medication. Randomised controlled trial. Br J Psychiatry 2005; 186:324–30.

31. van der Gaag M. CBT in persistent psychotic symptoms affects functioning and suffering. Eur Arch Psychiatry Clin Neurosci 2011; 261:S36-s37.

32. Wykes T, Hayward P, Thomas N, Green N, Surguladze S, Fannon D et al. What are the effects of group cognitive behaviour therapy for voices? A randomised control trial. Schizophr Res 2005; 77(2-3):201–10.
