# Supplementary material for: Response rates in patients with schizophrenia and positive symptoms receiving cognitive behavioural therapy: a systematic review and single-group meta-analysis
Source: BMC Psychiatry. 2018 Dec 4;18:380. doi: 10.1186/s12888-018-1964-8 (PMC6280425; doi:10.1186/s12888-018-1964-8)

Risk of bias assessment

Risk of bias summary

	Random sequence generation (selection bias)	Allocation concealment (selection bias)	Blinding of participants and personnel (performance bias)	Blinding of outcome assessment (detection bias)	Incomplete outcome data (attrition bias)	Selective reporting (reporting bias)	Researchers Allegiance	Other bias
Barrowclough 2006	+	+	−	+	−	+	?	+
Bechdolf 2004	+	?	−	+	−	−	+	+
Birchwood 2014	+	+	−	+	+	−	−	?
Drury 1996	?	?	−	−	−	?	?	+
Durham 2003	+	+	−	+	−	−	?	?
England 2007	+	?	−	?	?	?	−	+
Foster 2010	?	?	−	−	−	?	?	+
Freeman 2014	+	?	−	+	?	+	−	+
Freeman 2015a	+	?	−	+	−	−	−	+
Freeman 2015b	+	?	−	+	−	+	−	+
Garety 2008	+	+	−	+	−	+	−	+
Gottlieb 2017	?	?	−	?	−	+	−	+
Haddock 1999	?	?	−	?	−	?	−	+
Haddock 2009	+	?	−	+	−	?	?	+
Hazell 2016	?	+	−	?	?	?	−	?
Krakvik 2013	?	?	−	−	+	+	+	?
Kuipers 1997	?	?	−	−	−	−	−	?
Lecomte 2008	?	?	−	?	?	−	−	+
Lee 2012	?	?	−	?	−	?	?	+
Lee 2013 PT	?	?	−	?	−	?	?	+
Levine 1998	?	?	−	−	−	?	−	+
Li 2015	+	+	−	?	?	−	−	+
Morrison 2014	+	+	−	+	+	−	−	+
Penn 2009	+	?	−	+	?	?	−	+
Pinninti 2010	+	?	−	?	−	?	+	+
Rector 2003	?	?	−	+	−	+	−	?
Sensky 2000	?	?	−	?	?	−	−	+
Startup 2004	+	?	−	−	−	?	+	+
Tarrier 1998	?	?	−	+	+	−	−	?
Valmaggia 2005	+	?	−	?	−	?	−	?
van der Gaag 2011	?	?	−	?	−	−	?	+
Wykes 2005	?	?	−	−	−	?	−	+

Risk of bias graph

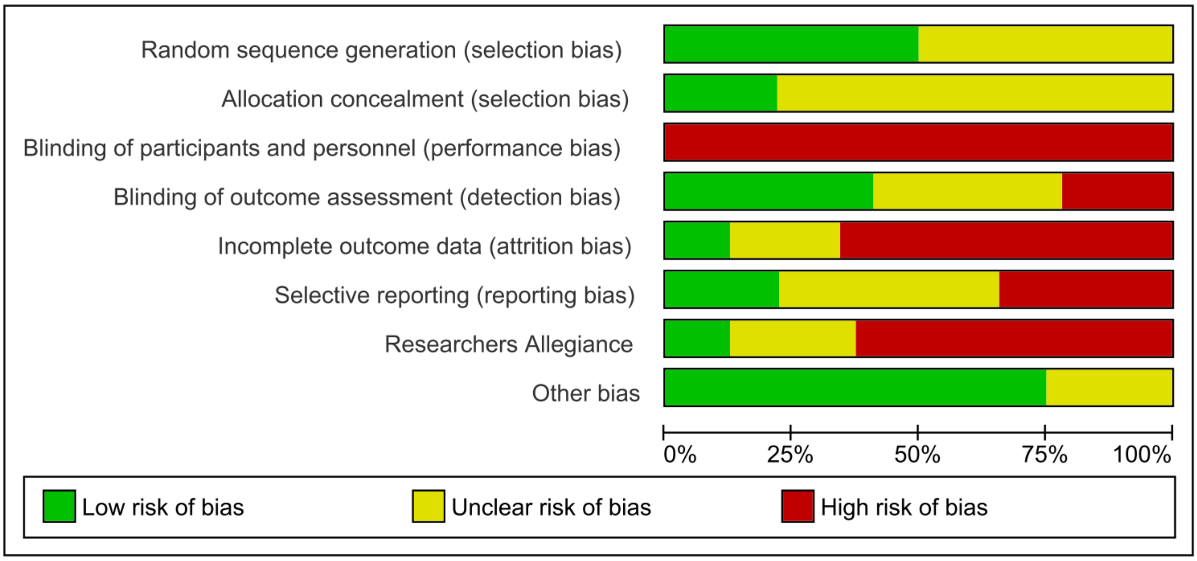

Supplement: Supplementary file 2 — Risk of bias assessment (PDF 766 kb) [file 12888_2018_1964_MOESM2_ESM.pdf]
