# Supplementary material for: Response rates in patients with schizophrenia and positive symptoms receiving cognitive behavioural therapy: a systematic review and single-group meta-analysis
Source: BMC Psychiatry. 2018 Dec 4;18:380. doi: 10.1186/s12888-018-1964-8 (PMC6280425; doi:10.1186/s12888-018-1964-8)
Supplement: Supplementary file 3 — Small study effect and publication bias (PDF 402 kb) [file 12888_2018_1964_MOESM3_ESM.pdf]

Investigation of small study effect and publication bias

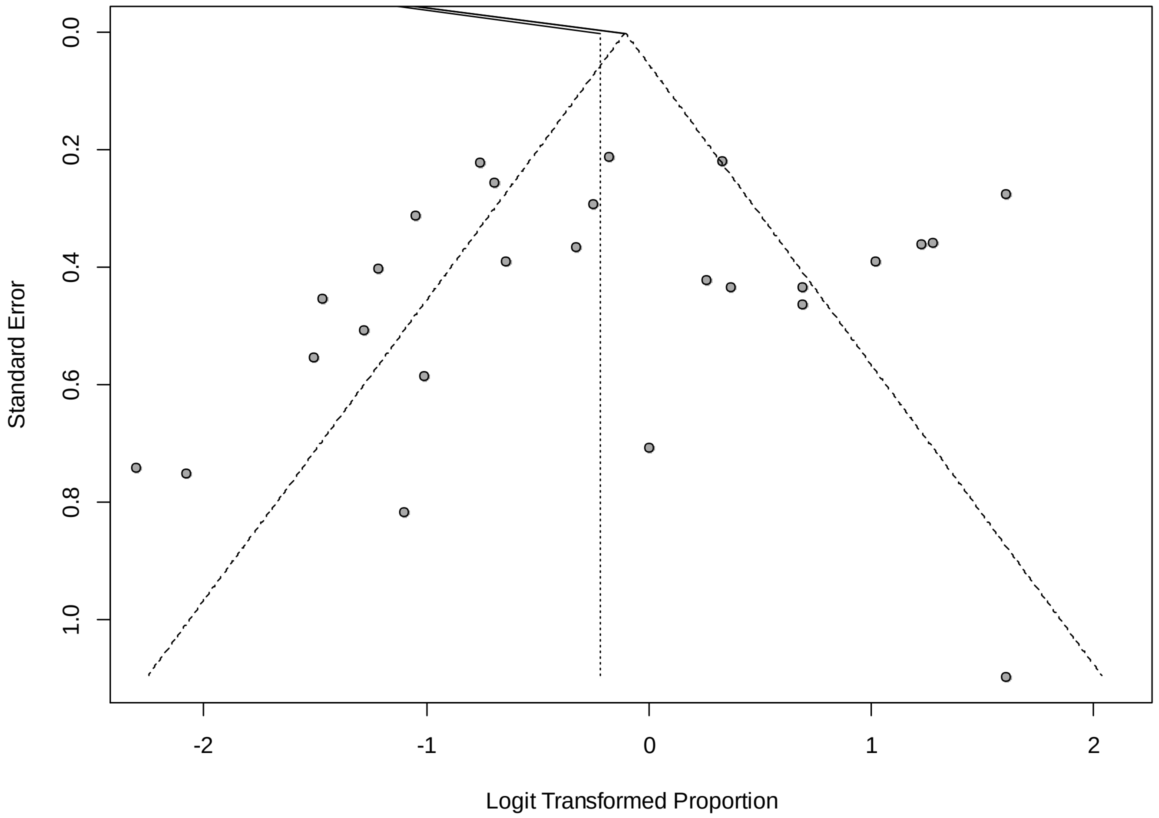

Funnel plot for primary outcome – response in overall symptoms, based on the 20% reduction from baseline threshold

Results of Egger's test for forest plot asymmetry:

Linear regression test of funnel plot asymmetry				
t = -0.82637, df = 24, p-value = 0.4167				
alternative hypothesis: asymmetry in funnel plot				
sample estimates:				
bias	se.bias	slope		
-1.1261606	1.3627763	0.2695401		
